# Stand from Under

**Published:** 1881-11-20

**Authors:** 


### *STAND FROM UNDER!*

The Eclectic Medical Journal, of Atlanta, in its October issue, seizing suddenly the sword of criticism and wrath, cuts blindly and furiously right and left, pitching into both "Regular" and "Irregular"—making huge and bloody gashes upon Scudder, of Cincinnati, the great leader and apostle in its own ranks.

